# Supplementary material for: Rapid wavefront shaping using an optical gradient acquisition
Source: Nat Commun. 2026 Jan 10;17:1537. doi: 10.1038/s41467-025-68259-2 (PMC12891611; doi:10.1038/s41467-025-68259-2)
Supplement: Supplementary file 1 — Supplementary Information [file 41467_2025_68259_MOESM1_ESM.pdf]

Rapid wavefront shaping using an optical gradient acquisition - supplementary material

Sagi Monin[1], Marina Alterman[1], Anat Levin[1]

[1]Department of Electrical and Computer Engineering, Technion, Haifa, Israel.

1 Optical system

System: A drawing of our system is shown in Fig. 1. A collimated laser passes through a polarizer to align the laser polarization with the SLM main-axis and is then expanded with a beam-expander (L1-L2). The wavefront is then modulated by the illumination SLM and reflected towards the sample. The modulated light passes through an objective lens and illuminates the sample. The forward scattered light continues to the validation camera, which is used for validating the focus of our algorithm on the image plane. The reflected light from the sample returns through the same objective lens and is reflected at a beam-splitter towards the imaging-SLM, which again modulates the wavefront. Finally, the light is collected by the main-camera sensor. The imaging-SLM has two functions: The first is to present the optimized pattern $\mathbf{u}^\ell(\boldsymbol{\rho})$ when measuring the score function and when confocal scanning a target. The second function is to present different defocus phase functions for capturing patterns for phase diversity optimization.

A full component list of our system: $532nm$ laser (CPS532 Thorlabs), P - linear polarizer (LPNIRB100 Thorlabs), L1 - $100mm$ achromatic lens, L2 - $400mm$ achromatic lens, L3 - $150mm$ achromatic lens, L4 - $100mm$ achromatic lens, L5 - $200mm$ achromatic lens, TL - tube lens (TTL200-A Thorlabs), BS - beamsplitter (62-882 Edmund), OL1 - objective lens NA=0.5, MAG=$\times$20 (N20X-PF Nikon), OL2 - objective lens NA=0.7, MAG=$\times$100 (MY100X-806 Mitutoyo). Lens translation stage is a single-axis stage (PT1 Thorlabs) with a motorized actuator (Z925B Thorlabs). The sample holder was custom created with three translation stages (PT1 Thorlabs) allowing to adjust the target in all three axes. For the main camera we use A314S Atlas (Lucid Vision) and for the validation camera we use Grasshopper3 USB3 (Teledyne Flir).

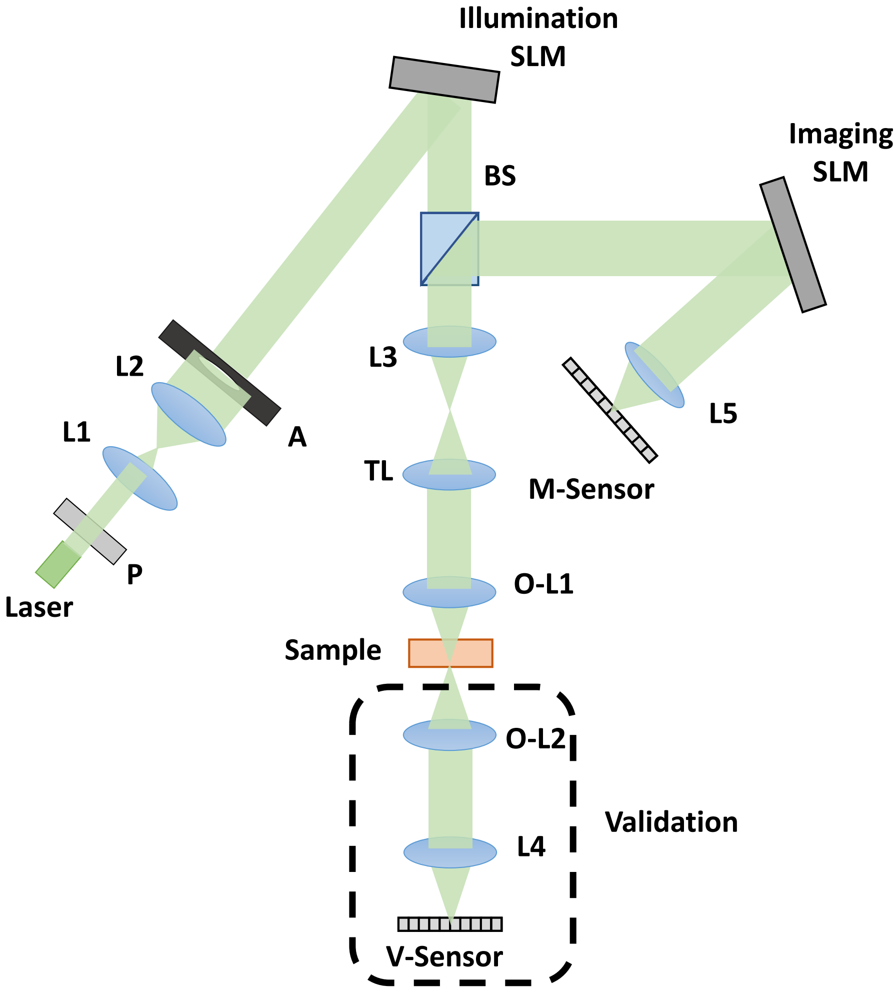

Fig. 1 **Setup:** we present the full setup of optical system. Light emitted by the laser is reflected from the illumination-SLM towards the sample. The reflected light from the sample returns through the same path where it is reflected from the imaging-SLM and is then collected by the main-sensor. The transmitted light from the sample continues to the validation sensor which is only used to validate our results. P:polarizer, BS:beam-splitter, L-lens, O-L:objective lens, V-sensor:Validation sensor, M-Sensor:Main sensor.

System alignment: We start by explaining how we align our system. To modulate the Fourier transform of the wave, the illumination SLM needs to be at the focal plane of the lens following it (L3 in Fig. 1), and the imaging-SLM at the focal plane of the lens before it (the same L3). We perform this alignment by using a third camera focused at infinity (we place an appropriate laser line filter ($532nm$) and focus the camera on a far building). Then we place two polarizers: before the SLM at a 45° angle and after the SLM at a 135° angle. Putting the polarizers in these angles converts the

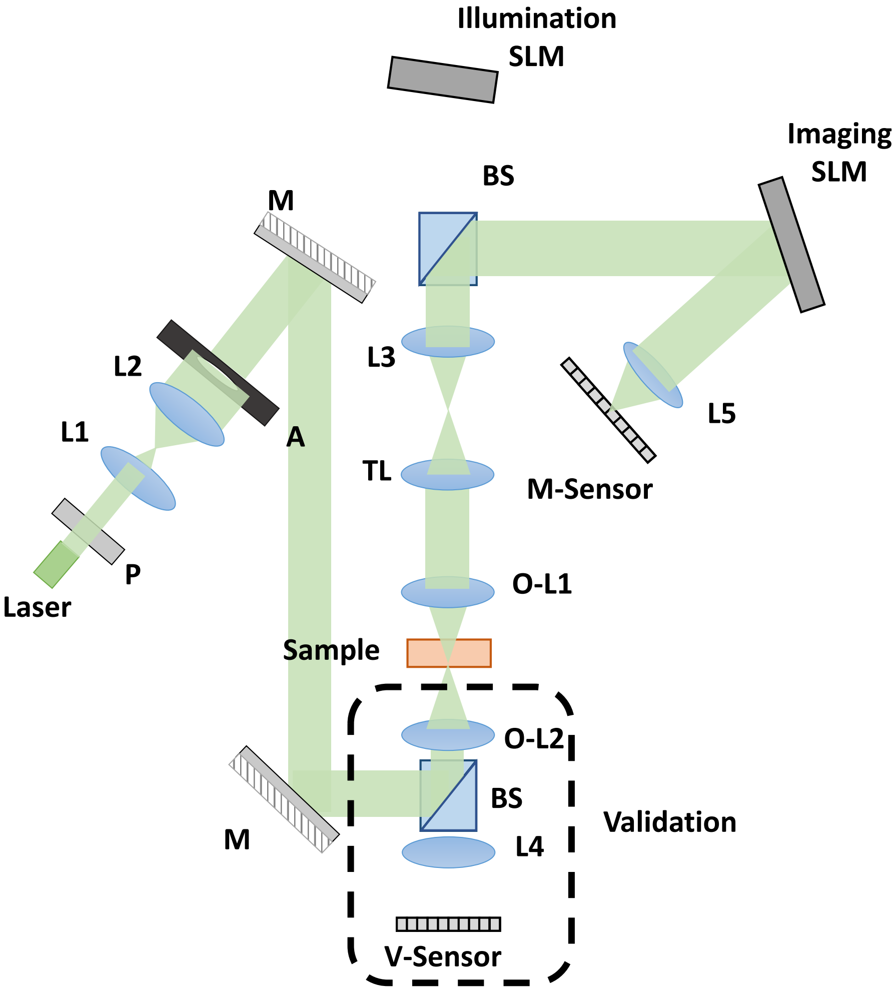

Fig. 2 Back calibration: We use a back-path laser to calibrate our system, here we show that laser path using the back-path.

phase modulation to intensity variation (a dark pixel of the SLM pixel if it does not modulate the light, and a white pixel if it modulates by π). We then focus on the SLM through the relevant lens, forming a relay system. We display a checkerboard pattern on the SLM and adjust the distance between the SLM and the lens until we get a sharp image. We also calibrate the distance between the main/validation cameras and the lenses before them (L4/L5) to ensure they are focused at infinity.

Finding the active SLM area: After the system is aligned, we identify the relevant illuminated areas on both SLMs that pass through the objective lens aperture. The size of these relevant areas can be calculated based on the aperture size and pixel size. We describe the calibration procedure for the illumination SLM; a similar procedure

is followed for the imaging-SLM. To find the center (x_c, y_c), we use the following procedure: We start by placing a mirror on the sample holder and moving the sample holder until we achieve focus at the main camera. To get an initial estimate of the centers we present a sinusoidal pattern of the same size as the aperture on the SLM and move it on $x - y$ axes of the SLM and look when we get a maximum intensity at the target pixel (if we do not modulate the right area on the SLM less light is directed to that target pixel), this gives us an initial estimate of (x_e, y_e). We then place a defocus pattern (quadratic phase $((x - (x_c - x_e))^2 + (y - (y_c - y_e))^2)$) on the illumination SLM and move the sample holder axially until again we achieve focus on the main camera. Ideally, if $(x_c, y_c) = (x_e, y_e)$ the location of the laser spot will be at the same $x - y$ location no matter what quadratic phase we introduce. If the location of the laser spot is different, we modify our estimation. We repeat this until the focus does not change. This is then repeated for the imaging-SLM while the illumination SLM presents a blank phase.

Mapping of SLM to camera: Next, we calibrate the mapping between the SLMs and the main camera. Using the focal length of the lens before the camera (L5), the SLM pitch, and the wavelength of the emitted light, we can map between SLM frequencies to the camera pixel using simple geometry. However, this mapping does not account for misalignment in the rotation of the SLMs. To overcome this, we fine-tune the calibration by displaying sinusoidal patterns and recording the shift presented on the main camera. Using these measurements, we estimate the rotation of the SLM.

Mapping between SLMs: Finally, we need to accurately map every pixel on the illumination SLM to the corresponding pixel on the camera SLM. To do this, we follow a similar procedure as in [1, 2]. However, instead of using a fluorescent bead, we illuminate the target from the back, bypassing the illumination SLM. That is, we place mirrors so the laser is directed through O-L2, and after the sample the light continues to the main camera, as shown in Fig. 2. We then recover the modulation pattern by applying phase-diversity optimization. The Helmholtz reciprocity (phase conjugation) principle [3] dictates that if we place the conjugate of the recovered wavefront on the illumination SLM, it will focus into a point behind the scattering tissue. Hence, we redirect the laser to the forward path Fig. 1 (i.e. through the illumination SLM and O-L1) and place the modulation pattern on illumination SLM. We then expect to achieve a focused point on the validation camera. However, if the mapping between the SLMs is incorrect, we do not get a focused point on the validation camera. We can then find the mapping between the SLMs by shifting the pattern on the illumination SLM until the energy of the focused point on the validation camera is maximal.

Determining target area size $\mathcal{A}$: As demonstrated in Fig. 4 in the paper, to focus light inside the tissue, we need to optimize our score over a target area $\mathcal{A}$ (also known as isoplanatic area). However, there is a compromise between the intensity of focus inside the tissue and the intensity of the focused spot on the main camera. In our work, to decide on the area size, we tried a few isoplanatic area sizes for each sample and kept the ones providing the best results. Once we found a good area for one isoplanatic window we could scan many windows in the same sample using the same $\mathcal{A}$ parameter and stitch them together. An alternative approach would be to use a

multi-scale $\boldsymbol{\mathcal{A}}$ size as presented in [4], where we start with a large area and decrease its size as we increase the iterations.

Tilt-shift parameter calibration: As our score maximizes the confocal intensity over an area, we rely on the tilt-shift memory effect. To apply the scan, we need to recover the parameter determining the ratio between the tilt and shift. To determine this parameter, we again follow a similar calibration method as in [1, 2]. However, since they rely on fluorescent beads, we instead illuminate the target from the back, bypassing the illumination SLM. We then recover the modulation pattern by applying phase-diversity optimization. We then redirect the laser to the forward path (i.e., through the illumination SLM and O-L1) and place the modulation pattern on the illumination SLM and we use the validation camera to view the focused spot. We then adjust the ratio between tilt and shift of the modulation pattern so that we can move the focused spot in the validation camera while preserving maximal intensity.

Gradient step: When performing gradient descent optimization, we need to choose a step-size at each iteration. In our system, we use backtracking line search [5]. For each iteration, we start with a predefined step size and after performing a step, we measure the score function. If the score function increases, we perform the step; however, if the score decreases, we do not perform the step. Instead, we divide the step size by two and again measure the score. We repeat this until the score increases or until the step size is smaller than some threshold. If the score does not increase, we perform a large step (with the initial step size) as the optimization might be stuck in a local maximum. At the end of optimization, we choose the phase modulation pattern that scored the highest.

2 Justifying the confocal area score

To find a good wavefront shaping modulation, our algorithm relies on a score function that measures the confocal intensity over a small area $\boldsymbol{\mathcal{A}}$. To do this, we scan the area by tilting and shifting the modulation toward a point $\ell \in \boldsymbol{\mathcal{A}}$ and collect the averaged confocal intensity:

$$\mathcal{S}(\boldsymbol{\rho}) \equiv \sum_{\ell \in \boldsymbol{\mathcal{A}}} |\mathbf{u}^{\ell}(\boldsymbol{\rho})^T \boldsymbol{\mathcal{R}} \mathbf{u}^{\ell}(\boldsymbol{\rho})|^2. \tag{1}$$

This score was used by several recent approaches for digital aberration correction [6–8]. They optimize over finite isoplanatic patches to maximize the diagonal elements of the reflection matrix, and the sum of these diagonal elements is equivalent to our confocal area score. To better explain why this score favors focusing wavefront-shaping modulations, we provide a derivation below. For that, let us denote by $\boldsymbol{\mathcal{T}}$ the transmission matrix describing the propagation of light from the SLM plane to a mirror attached at the back of the scattering volume. It has been shown that the reflection matrix can be expressed as a double pass through the transmission matrix [6, 7, 9]

$$\boldsymbol{\mathcal{R}} = \boldsymbol{\mathcal{T}}^T \cdot \boldsymbol{\mathcal{T}}, \tag{2}$$

where $\boldsymbol{\mathcal{T}}^T$ is the transpose of the transmission matrix (this is just transpose, not a conjugate transpose).

We now consider an incoming modulation $\mathbf{u}^{\ell}(\boldsymbol{\rho})$ and denote the complex wavefront it generates at the back of the tissue as

$$\boldsymbol{\nu}^{\ell} = \boldsymbol{\mathcal{T}}\mathbf{u}^{\ell}(\boldsymbol{\rho}). \tag{3}$$

We will express the confocal area score using the wavefront at the back layer and show that under idealized memory effect correlation, this score is equivalent to the nonlinear score of two-photon fluorescent excitation [10], or the single photon confocal fluorescent feedback [1]. It has already been shown that such a score is maximized by focusing modulations, namely, when $\boldsymbol{\nu}$ is a one-hot vector that brings all the energy into a single point and has zero intensity over the rest of its entries.

Claim 1. *Let $\boldsymbol{\nu}^{o} = \boldsymbol{\mathcal{T}}\mathbf{u}^{o}(\boldsymbol{\rho})$ denote the wavefront at the back of the tissue where the incoming illumination is directed to a point ℓ_o at the center of the patch $\boldsymbol{\mathcal{A}}$. If memory effect correlation holds over the area $\boldsymbol{\mathcal{A}}$, the confocal area score of Eq. (12) reduces to:*

$$\mathcal{S}(\boldsymbol{\rho}) = \sum_{x} |\boldsymbol{\nu}^{o}(x)|^{4}, \tag{4}$$

where x is a 2D position vector, running over the coordinates of $\boldsymbol{\nu}^{o}$.

Proof. The confocal intensity resulting from one modulation tilted toward point ℓ can be expressed as:

$$I^{\ell}(0) = \left| \sum_{x} (\boldsymbol{\nu}^{\ell}(x))^{2} \right|^{2}, \tag{5}$$

where x runs over points on the back plane of the tissue, and at each such point we square the complex field while maintaining its phase (this is not an absolute value). The confocal area score can then be expressed as:

$$\mathcal{S}(\boldsymbol{\rho}) = \sum_{\ell} I^{\ell}(0) = \sum_{\ell} \left| \sum_{x} (\boldsymbol{\nu}^{\ell}(x))^{2} \right|^{2} \tag{6}$$

We now use the tilt-shift memory effect [11, 12] that states that if we tilt the incoming modulation towards two points ℓ and ℓ_o (where ℓ_o denotes the center of the patch, and ℓ is another point in $\boldsymbol{\mathcal{A}}$) separated by a 2D displacement vector $\Delta_{\ell} = \ell - \ell_o$, then the fields at the back of the tissue satisfy a tilt-shift correlation and

$$\boldsymbol{\nu}^{\ell}(x) \approx \boldsymbol{\nu}^{o}(x + \Delta_{\ell}) e^{\frac{2\pi i}{\lambda L}(\Delta_{\ell} \cdot x)}, \tag{7}$$

where L is $2/3$ of the tissue thickness. Assuming the memory effect correlation is strong enough, we can use the wavefront $\boldsymbol{\nu}^{0}$ generated when directing light to the center of

the patch $\mathcal{A}$, and express all other wavefronts ν^ℓ, generated when directing light to the rest of the patch. With this, we can express the confocal area score of Eq. (6) as

$$\mathcal{S}(\rho) = \sum_\ell \left| \sum_x (\nu^o(x))^2 e^{\frac{2\pi i}{\lambda L}(\Delta_\ell \cdot x)} \right|^2. \tag{8}$$

Expanding Eq. (8) we can write

$$\mathcal{S}(\rho) = \sum_{x_1,x_2} (\nu^o(x_1))^2 \cdot (\nu^o(x_2)^*)^2 \sum_\ell e^{\frac{2\pi i}{\lambda L}(\Delta_\ell \cdot (x_1 - x_2))} \tag{9}$$

We now note that if $x_1 \neq x_2$ and the range of Δ_ℓ values is large enough terms of the form $\sum_\ell e^{\frac{2\pi i}{\lambda L}(\Delta_\ell \cdot (x_1 - x_2))}$ are equivalent to the mean of a sinusoidal, which is 0. Therefore, Eq. (10) reduces to:

$$\mathcal{S}(\rho) = \sum_x |\nu^o(x))|^4, \tag{10}$$

as desired.□

Claim 2. *Assuming sufficient memory effect correlation, the confocal area score is maximized when the incoming modulation ρ makes the wavefront ν at the back of the tissue a sparse one-hot vector.*

Proof. Using the previous claim, the confocal area score is equivalent to $\mathcal{S}(\rho) = \sum_x |\nu^o(x)|^4$. This is equivalent to the confocal score measured using incoherent fluorescent wavefront shaping [1] with single photon excitation, or to the nonlinear emission using two photon excitation [10]. It has already been shown that this score favors focusing modulations. To see this, note that the total laser power is bounded and by using a modulation, we can spread the light in different ways, but we cannot increase its power. Therefore, for any modulation ρ the norm of the wavefront ν^o behind the tissue is bounded.

$$\sum_x |\nu^o(x)|^2 \leq C. \tag{11}$$

It is easy to see that to maximize $|\nu^o(x)|^4$ under the bounded norm constraint, it is best if ν^o is a one-hot vector that has all its power in one entry and zero energy at all other entries. □

Tilt-shift and time reversal: Our algorithm optimizes the wavefront to ensure that input and output fields are similar and exhibit the memory effect and ensure that focusing on adjacent points results in tilt-shifted versions of each other. To illustrate this, we present simulation results of our algorithm in Fig. 3. Assuming the SLM is conjugate to the output plane, we demonstrate the focusing achieved for two spots inside the tissue before and after applying our algorithm.

We simulate a scenario without applying wavefront correction and present the intensity on the imaging plane. In this case, light is aberrated and spread across the

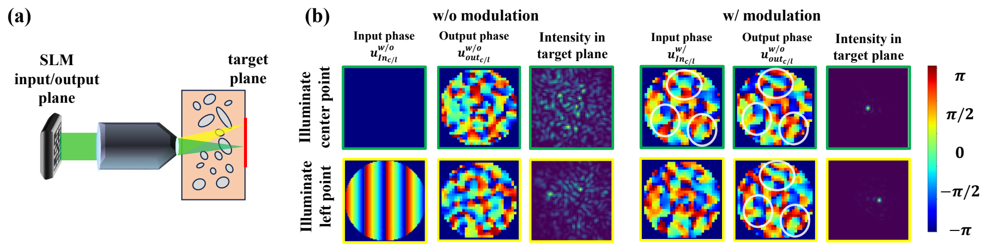

Fig. 3 Tilt-shift and time reversal effects: (a) We show a simplified schematic of our system with two input wavefronts, green focusing into the center of the imaging plane and yellow focusing to a neighboring point to the left. (b) Simulation results showing the incoming phase on the SLM, the conjugate of the output phase after tissue reflection (when reaching the SLM plane), and the intensity at the target plane. With light modulation, strong correlations are observed between the incoming SLM phase and the output phase, as well as between output phases for different points (white circles indicate areas of strong correlation). Without aberration correction, correlation decreases rapidly.

target plane. This results in an uncorrelated reflected wavefront returning to the SLM plane $u^{w/o}_{out}$, when compared with the conjugate phase presented on the SLM $u^{w/o}_{in}$, with correlation score: $\boldsymbol{C}\left(u^{w/o}_{in}, u^{w/o}_{out}\right) = 0.51$. Additionally, since light reflects from a large area inside the tissue, wavefronts reflected from two different points $u^{w/o}_{out_c}, u^{w/o}_{out_l}$ are also uncorrelated, with $\boldsymbol{C}\left(u^{w/o}_{out_c}, u^{w/o}_{out_l}\right) = 0.47$.

Conversely, after applying our algorithm to optimize a wavefront that incorporates both memory effect and time-reversal principle, we achieve focus on the target plane. This improvement is exhibited by increased correlation between incoming SLM modulation and output wavefronts $\boldsymbol{C}\left(u^{w/}_{in}, u^{w/}_{out}\right) = 0.87$, as well as enhanced correlation between wavefronts reflected by neighboring points on the target plane $\boldsymbol{C}\left(u^{w}_{out_c}, u^{w/}_{out_l}\right) = 0.77$.

3 Gradient acquisition

3.1 Score and gradient with definitions

We seek to maximize the confocal intensity averaged over a target area $\boldsymbol{\mathcal{A}}$, where $\boldsymbol{\rho}$ is a complex wavefront displayed on the SLM:

$$\mathcal{S}(\boldsymbol{\rho}) \equiv \sum_{\ell \in \boldsymbol{\mathcal{A}}} |\mathbf{u}^{\ell}(\boldsymbol{\rho})^T \boldsymbol{\mathcal{R}} \mathbf{u}^{\ell}(\boldsymbol{\rho})|^2. \tag{12}$$

Differentiating this score with respect to $\boldsymbol{\rho}$ provides:

$$\frac{\partial \mathcal{S}(\boldsymbol{\rho})}{\partial \boldsymbol{\rho}} = 2 \sum_{\ell} \underbrace{\left(\mathbf{u}^{\ell}(\boldsymbol{\rho})^T \boldsymbol{\mathcal{R}} \mathbf{u}^{\ell}(\boldsymbol{\rho})\right)^*}_{(1)} \cdot \underbrace{\left(\boldsymbol{\mathcal{R}} \mathbf{u}^{\ell}(\boldsymbol{\rho}) + (\mathbf{u}^{\ell}(\boldsymbol{\rho})^T \boldsymbol{\mathcal{R}})^T\right)}_{(2)} \odot \underbrace{\frac{\partial \mathbf{u}^{\ell}(\boldsymbol{\rho})}{\partial \boldsymbol{\rho}}}_{(3)}. \tag{13}$$

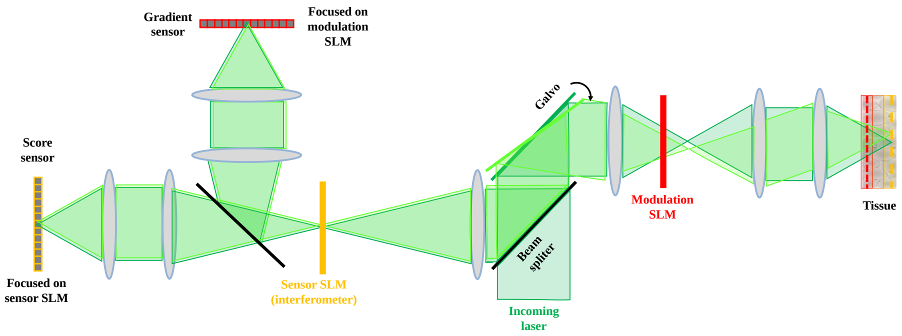

Fig. 4 Setup for interferometric gradient acquisition: An incoming laser beam is directed to focus on a point inside the tissue, the focal point can be shifted with a galvo. The figure illustrates two such tilted paths in two shades of green. On its way to the sample the beam passes through a relay system, containing an SLM for aberration correction. The SLM is placed such that it is conjugate to a plane inside the tissue volume. Another SLM is used as an interferometer to modulate the reflected light captured by the sensor. Two cameras are used in this setup - the first is focused on the interferometer SLM and termed score sensor. The second sensor is focused on the modulation SLM and termed gradient sensor.

In most SLMs we can only adjust the phase of the wavefront while its amplitude is fixed, so $\boldsymbol{\rho}$ is a wavefront of the form $\boldsymbol{\rho}(x) = e^{2\pi\boldsymbol{\theta}(x)}$. To further differentiate the score with respect to $\boldsymbol{\theta}$ one can use the chain rule once more, leading to

$$\frac{\partial \mathcal{S}(\boldsymbol{\rho})}{\partial \boldsymbol{\theta}} = 2\pi Im\left(\boldsymbol{\rho} \odot \frac{\partial \mathcal{S}(\boldsymbol{\rho})}{\partial \boldsymbol{\rho}}\right) \tag{14}$$

Where Im denotes the imaginary part of the complex wavefront.

3.2 Gradient acquisition using phase diversity optimization

To acquire the gradient of our wavefront shaping score, we need to capture the complex wavefront arriving at the sensor $\mathbf{v}^\ell = \boldsymbol{\mathcal{R}}\mathbf{u}^\ell(\boldsymbol{\rho})$, for every point ℓ in the scanned area $\boldsymbol{\mathcal{A}}$. In our current implementation, we do this using a phase diversity optimization scheme [13, 14] for each of the target points. That is, we sequentially place the modulation wavefronts $\mathbf{u}^\ell(\boldsymbol{\rho})$ on the illumination SLM and capture the resulting speckle intensity images at the sensor. We place on the camera SLM five known defocus wavefronts $\mathbf{u}^j_{\text{defocus}}$ and capture five speckle intensity images $I^1_\ell, \dots I^5_\ell$. We use these images to solve an optimization problem to to retrieve the phase of the complex wavefront from the measured intensity images [15] resulting in the optimization problem:

$$\hat{\mathbf{v}}^\ell = \arg\max_{\mathbf{v}^\ell} \sum_{j=1}^{5} \left\| I^j_\ell - |\boldsymbol{\mathcal{F}}(\mathbf{v}^\ell \odot \mathbf{u}^j_{\text{defocus}})|^2 \right\|^2 . \tag{15}$$

We scan multiple points $\ell \in \boldsymbol{\mathcal{A}}$ and repeat the same phase optimization. These wavefronts are averaged to compute one instance of the gradient.

Note that as long as the magnification of the imaging system is set such that the speckle grain of the captured image I^j is larger than a pixel, there is no inherent limitation on the resolution of the recovered wavefront $\mathbf{v}^\ell$.

3.3 Fast gradient acquisition using point diffraction interferometry

We describe another future approach for gradient acquisition which would allow us to acquire it much faster, using a small number of only three shots. This possible setup would utilize a variant of a point diffraction interferometry system [16, 17] that can capture the complex wavefront with no optimization, and can also average over all $\ell \in \boldsymbol{\mathcal{A}}$ within one shot.

Setup for point diffraction interferometry: Fig. 4 illustrates a setup for gradient acquisition with point diffraction interferometry. A laser beam illuminates a tissue sample, and an SLM can modulate its shape. The SLM is placed such that it is conjugate to a plane inside the tissue, where the aberration is actually happening. The illumination wavefront propagates through the scattering tissue, reflects from its internal structure, and back-scatters to the camera. The returning light passes through the same SLM on its way to the camera. The returning modulated light splits at a beam splitter toward two sensors. Before the sensors, the light passes through another relay system and a second SLM. This second SLM is placed such that it is conjugate to the target we wish to image, rather than conjugate to the aberration plane. We refer to the first SLM (closer to the sample) as "the modulation SLM" and to the second one as "the sensor SLM". After the sensor SLM, the light splits through another beam splitter toward two sensors. The first sensor (which can also be a single point detector) is placed such that it is conjugate to the target points we wish to image and to the sensor SLM, and would measure the confocal intensity score at the center of the frame. The second 2D sensor is placed such that it is conjugate to the modulation SLM and would measure the gradient directly. We refer to these two sensors as the *score sensor* and the *gradient sensor*.

The setup also includes a galvo mirror that can tilt the incoming and outgoing light. While we can implement such a tilt with the SLM itself, a galvo mirror can be tilted faster than the refresh rate of the SLM. By tilting the incoming beam, we can direct it toward different target points $\ell \in \boldsymbol{\mathcal{A}}$. Tilting the returning beam implies that the light scattered from target point ℓ is directed to the central pixel of the sensor, regardless of the actual spatial position of ℓ. In this setup, to illuminate the tissue with the modulation wavefront $\mathbf{u}^\ell(\boldsymbol{\rho})$, we place $\boldsymbol{\rho}$ on the SLM and use the galvo to tilt it toward the target point ℓ. So, effectively we get:

$$\mathbf{u}^\ell(\boldsymbol{\rho})(x) = \boldsymbol{\zeta}_x \odot e^{\frac{2\pi i}{\lambda}(\tau^\ell \cdot x)} \odot \boldsymbol{\rho}(x), \tag{16}$$

where τ^ℓ represents the tilt toward point ℓ. $\boldsymbol{\zeta}_x$ represents here the shape of the illumination wavefront reaching the modulation SLM. Since our SLM is not placed at the Fourier plane of the system, it is illuminated by a spherical wavefront and not by a plane wave. Note that $\boldsymbol{\zeta}_x$ was defined in the main paper as the (known) propagation of the wavefront from the modulation SLM to the center of the sensor. Since we assume

our system is fully symmetric the same $\boldsymbol{\zeta}_x$ applies both on the incoming and on the outgoing paths.

Interferometric measurements: We start with a mathematical expression for the wavefront we will measure by the gradient sensor. We recall that this sensor is focused at the modulation SLM and directly images the wavefront at this plane. If we use a flat phase on the sensor SLM and display the modulation $\mathbf{u}^\ell(\boldsymbol{\rho})$ (by displaying $\boldsymbol{\rho}$ on the modulation SLM and combining it with the proper galvo tilt), it will measure the 2D wavefront:

$$\mathbf{v}^\ell = \left(\mathbf{u}^\ell(\boldsymbol{\rho})\right) \odot \left(\boldsymbol{\mathcal{R}}\mathbf{u}^\ell(\boldsymbol{\rho})\right), \tag{17}$$

where $\boldsymbol{\mathcal{R}}\mathbf{u}^\ell(\boldsymbol{\rho})$ is the propagation of the incoming modulated illumination via the tissue and back to the SLM plane. On the SLM plane, each coordinate is multiplied again by $\mathbf{u}^\ell(\boldsymbol{\rho})$. We use the notation $\odot$ to denote an element-wise multiplication between two 2D fields. Since we image the modulation SLM plane, we measure this as a 2D wavefront and not as a scalar.

Next, denote by μ^ℓ the complex scalar reaching the center of the score sensor when there is a blank phase on the sensor SLM. As this sensor is focused on the target plane (and on the sensor SLM, which is conjugate to the target plane):

$$\mu^\ell = (\mathbf{u}^\ell(\boldsymbol{\rho}))^T \boldsymbol{\mathcal{R}}\mathbf{u}^\ell(\boldsymbol{\rho}), \tag{18}$$

where $\mathbf{u}^\ell(\boldsymbol{\rho})$ is defined in Eq. (16) to include the SLM modulation, the tilt, and the propagation from the modulation plane to the score sensor.

If we could place an aperture on pixel x of the 2nd sensor SLM (so we block the parts of the wavefront that do not pass via point x), the 2D wavefront we measure at the gradient sensor (which is focused on the modulation SLM) is governed by the scalar μ^ℓ and corresponds to:

$$\mathbf{v}_1^\ell = \mu^\ell \cdot {\boldsymbol{\zeta}_x}^*, \tag{19}$$

where $\boldsymbol{\zeta}_x$, defined in Eq. (2) of the main paper, encodes the (known) propagation from the modulation SLM plane to the center of the score sensor.

We denote the difference between the wavefronts in Eqs. (17) and (19) by:

$$\mathbf{v}_2^\ell = \mathbf{v}^\ell - \mathbf{v}_1^\ell. \tag{20}$$

Moreover, rather than using a pinhole on the sensor SLM, we suggest varying the phase of pixel x of the sensor SLM to ϕ_j while keeping all other pixels at phase 0. This implies that the gradient sensor would see the wavefront $\mathbf{v}_2^\ell + e^{i\phi_j}\mathbf{v}_1^\ell$, where $\mathbf{v}_2^\ell$ is the part of the wavefront that passes when blocking pixel x, and $e^{i\phi_j}\mathbf{v}_1^\ell$ is the part that passed via pixel x after its phase is adjusted. We can use standard phase shifting interferometry and measure $J = 3$ intensity images while varying the phase ϕ_j of the central pixel equally between 0 and 2π. We measure

$$I_j^\ell = |\mathbf{v}_2^\ell + e^{i\phi_j}\mathbf{v}_1^\ell|^2 = e^{-i\phi_j}\mathbf{v}_1^{\ell^*}\mathbf{v}_2^\ell + e^{i\phi}\mathbf{v}_1^\ell\mathbf{v}_2^{\ell^*} + |\mathbf{v}_1^\ell|^2 + |\mathbf{v}_2^\ell|^2. \tag{21}$$

By summing the intensity images with the corresponding phase, we can isolate the interference term

$$\mathbf{v}_1^{\ell *}\mathbf{v}_2^{\ell} = \sum_j e^{i\phi} I_j^{\ell}. \tag{22}$$

We can also measure $|\mathbf{v}_1^{\ell}|^2$ and add it to Eq. (22) to receive

$$g^{\ell} = \mathbf{v}_1^{\ell *}\mathbf{v}^{\ell}. \tag{23}$$

We can now substitute Eqs. (16), (17) and (19), and check the content of this interference term:

$$g^{\ell} = \mu^{\ell *} \cdot \left(\boldsymbol{\zeta}_x \odot \mathbf{u}^{\ell}(\boldsymbol{\rho})\right) \odot \left(\boldsymbol{\mathcal{R}}\mathbf{u}^{\ell}(\boldsymbol{\rho})\right) \tag{24}$$

$$= \underbrace{\mu^{\ell *}}_{(1)} \cdot \underbrace{\boldsymbol{\zeta}_x \odot \boldsymbol{\rho}}_{(2)} \odot \underbrace{\boldsymbol{\zeta}_x \odot e^{\frac{2\pi\imath}{\lambda}(\tau^{\ell}\cdot x)}}_{(3)} \odot \underbrace{\left(\boldsymbol{\mathcal{R}}\mathbf{u}^{\ell}(\boldsymbol{\rho})\right)}_{(4)}. \tag{25}$$

Comparing this formula to the gradient derived in Eq. (13), we see that it corresponds to the desired gradient up to known multiplicative terms. Term (1) corresponds to term (1) of the gradient, term (2) is a known multiplicative factor, term (3) is effectively the derivative of the tilted modulation $\mathbf{u}^{\ell}(\boldsymbol{\rho})$ with respect to the SLM parameters $\frac{\partial \mathbf{u}^{\ell}(\boldsymbol{\rho})}{\partial \boldsymbol{\rho}}$, which is term (3) in Eq. (13), see definition in Eq. (16). Term (4) is equivalent to term (2) in Eq. (13).

Moreover, note that to average over target points $\ell \in \boldsymbol{\mathcal{A}}$, all we need to do is to scan the galvo toward a different direction, without changing the pattern $\boldsymbol{\rho}$ on the SLM. This effectively implies that we can tilt the galvo *within exposure*, and capture three speckle images

$$I_j = \sum_{\ell} I_j^{\ell}. \tag{26}$$

From these three images we can compute the gradient directly.

The above derivation suggests a possible future implementation of our wavefront shaping algorithm which will allow us to image the gradient of the confocal score directly, using as little as three shots; where the scanning over the target area is done within exposure. Furthermore, since we use an interferometer, no phase diversity optimization is required, and the complex wavefront is measured directly.

4 Equivalence to power iterations

The gradient derived above is similar to rapid time reversal based approach for wavefront shaping [2, 18–22]. Similar ideas were also used by digital correction algorithms [4, 6, 23]. These approaches start from the assumption that the desired modulation is the largest eigenvector of the reflection (or transmission) matrix of the tissue. This eigenvector can be estimated very efficiently using power iterations. In each iteration, one displays a modulation $\mathbf{u}$ on the SLM, measures $\boldsymbol{\mathcal{R}}\mathbf{u}$, and uses it as the next guess for the modulation.

We argue that the gradient we derived above collapses to such a power iteration if we restrict our score function to an area $\boldsymbol{\mathcal{A}}$ consisting of a single target point ℓ. To see this, note that if we attempt to maximize the score

$$\mathcal{S}(\boldsymbol{\rho}) = \left| \mathbf{u}^{\ell}(\boldsymbol{\rho})^{T} \boldsymbol{\mathcal{R}} \mathbf{u}^{\ell}(\boldsymbol{\rho}) \right|^{2}, \tag{27}$$

under the constraint that $\boldsymbol{\rho}$ is a unit norm vector (since the SLM can only redistribute the laser energy but it cannot regenerate energy), then classical results in linear algebra imply it is maximized by the largest eigenvector of $\boldsymbol{\mathcal{R}}$. Moreover, up to known constants, the gradient derived in Eq. (24) is basically a multiplication of the previous guess by the reflection matrix. However, as demonstrated in Fig. 4 of the main paper, given a coherent target, averaging over an area is crucial for getting a good wavefront correction. If one attempts to optimize the confocal intensity at a single point, interference can bring all light into a strong point at the sensor, without actually focusing the light into a single point inside the volume.

Attempting to adjust power algorithms to produce different modulations may require ad-hoc additions. However, defining a target score and deriving its gradient provides a more principled framework to impose desired properties on the modulation.

In particular [2] used a variant of power iterations with incoherent fluorescent sources. The convergence of this incoherent version was a bit hard to analyze, and to justify it the supplementary file of [2] uses a model assuming the phase of the wavefront is captured precisely with a point diffraction interferometer. Their incoherent phase estimation is equivalent to the point-diffraction interferometry setup derived above.

5 Additional results

Additional result of beads:

We use our system to image polystyrene beads dispersed in agarose gel. We create two different targets, with bead diameters of $0.5\mu m$ and $3\mu m$. The slab thickness is $1.3mm$. The left part of Fig. 5 illustrates the schematic of this target, where we used our algorithm to focus inside this volume and image planar sub-regions at multiple depths. The optical depth (OD) is 3, where OD was estimated by measuring the attenuation of ballistic light in the validation camera.

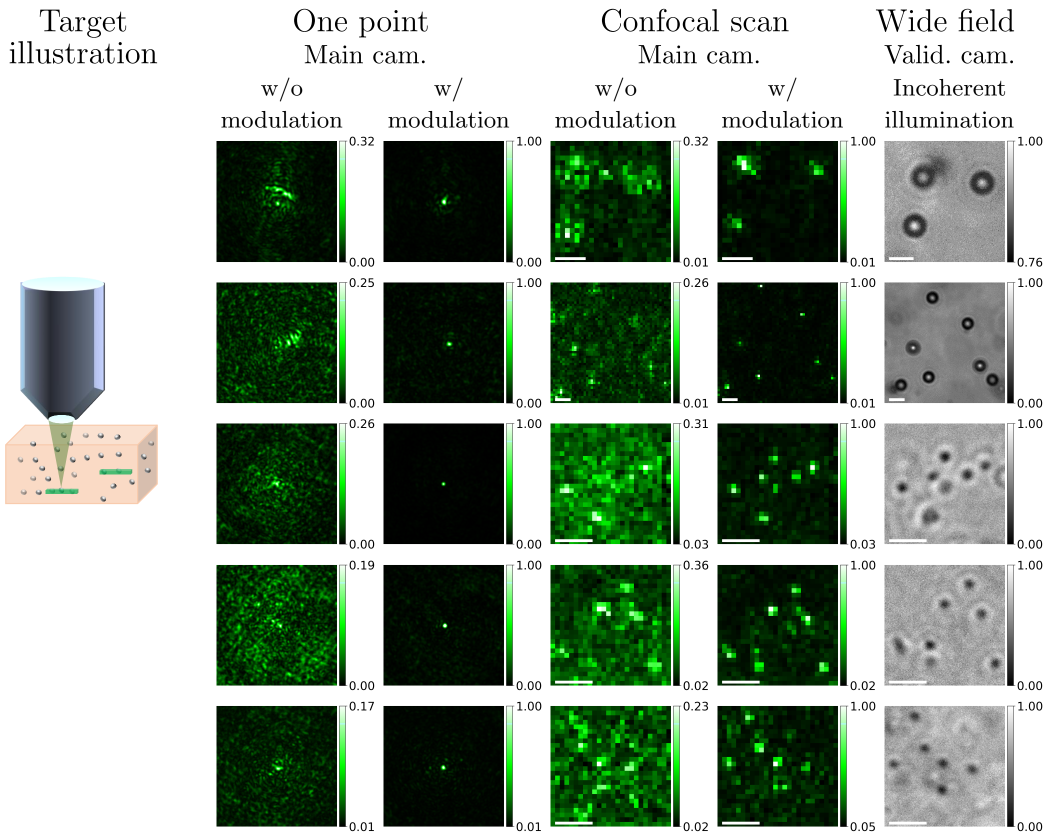

Fig. 5 Confcal scan of beads: Our algorithm used to image a scattering dispersion of polystyrene beads in agarose gel. The first column depicts a schematic view of the target, where we image small regions in different spatial areas and varying depth of the gel. The green bars inside the target depict the different areas which were imaged. Columns 2-3: Main camera images of a single focal spot before and after correction. Columns 4-5: Confocal scanning results: with and without aberration correction. Column 6: A reference image of the target captured from the validation camera behind the target, under wide-filed incoherent illumination. Rows 1-2 use $3\mu m$ diameter beads and rows 3-5 use $0.5\mu m$ diameter beads. Scale bar on confocal images is $4\mu m$. Note that an ideal aberration free image of beads in a reflection mode confocal microscope is a diffraction limited spot [24], even if the beads are larger than the diffraction limit since they act as a mini-lens focusing light.

Fig. 5 visualizes confocal scanning of $x-y$ slices, and Fig. 6 visualizes a slice along the $x-z$ axes. While a standard confocal scan of such beads is very noisy, with our estimated modulation, we can largely increase its contrast and achieve a clear image of spots corresponding to the beads position. To obtain a reference image of the bead positions, we image beads at the farther depth of the slab closer to the validation camera, so we are able to image a clear reference from the validation camera. For this reference image, we used wide-field incoherent illumination.

For the bead targets in Fig. 5, since the beads are sparser, we vary the target area, so we image multiple beads. The target area for the $3\mu m$ beads in the first two rows was $\boldsymbol{\mathcal{A}} = 16\mu m \times 16\mu m$ and $\boldsymbol{\mathcal{A}} = 32\mu m \times 32\mu m$, respectively. The sampling interval was $0.65\mu m$ for both algorithm and final confocal scan. For the $0.5\mu m$ beads (rows 3-5) the area was $\boldsymbol{\mathcal{A}} = 13\mu m \times 13\mu m$ with sampling interval of $0.5\mu m$ for both the algorithm and the final scan. In Fig. 6 the area was $\boldsymbol{\mathcal{A}} = 5.2\mu m \times 16\mu m$, the sampling interval in lateral axis was $0.5\mu m$ and sampling interval in axial was $2\mu m$. We note that even if the beads are larger than the diffraction limit the expected non-aberrated image of the bead in a reflection confocal scanning is a diffraction-limited spot, since the beads act as a mini-lens [24]. Consequently, $3\mu m$ beads appear as narrow isolated spots after our correction is applied.

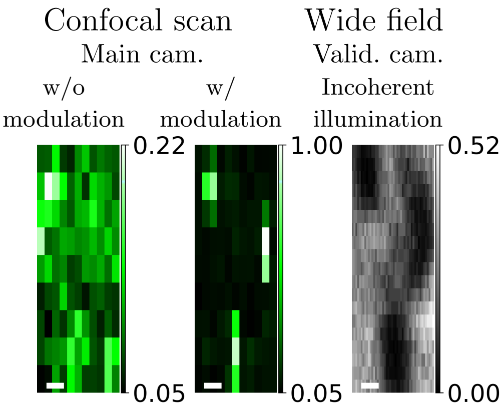

Fig. 6 Confocal scan of beads, $x-z$ slice: We apply our algorithm on a 2D $x-z$ slice, by utilizing the memory effect on the lateral axis and add quadratic phase to change focusing plane in the axial direction. We image an area $\boldsymbol{\mathcal{A}} = 5.2\mu m \times 16\mu m$. The scale bar is $1.2\mu m$.

Confocal scan captured images:

In Fig. 7, we display a super-position of images captured by the main and validation cameras. We compose in one frame a sparse subset of the target points ℓ in the area we scanned. We show the results before and after optimization for the result presented in the second row of Fig. 5 of the main text. Before optimization, the wavefront reaching the main camera is significantly aberrated. After optimization, clear focal points reflected from the chrome are observed. In the validation camera, we observe the wavefront reaching the target plane. Without modulation, the wavefront is highly aberrated, whereas after optimization, a well-defined focus is visible on the target plane. Areas covered by chrome are not observable in the validation camera because the chrome attenuates light. For visualization purposes, all images were normalized by their maximum value. However, in reality, without modulation, the light reaching both cameras is scattered and spread across the sensor, and these images are much darker than the images captured using our modulation.

The resolution target in Fig. 5 in the main paper is constructed from four scanned isoplanatic patches. In Fig. 8, we show the different confocal scans from the different isoplanatic patches and their corresponding phase masks.

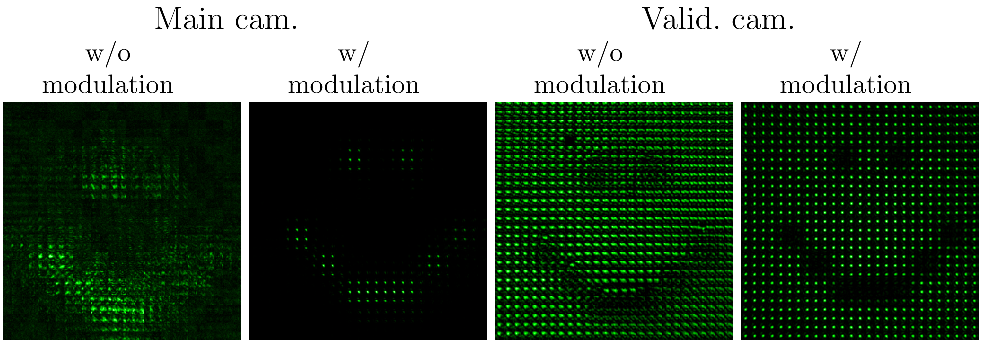

Fig. 7 **Individual scanning images:** We present the scanning results before and after optimization from the Main camera and Validation camera. Before optimization the light is scattered at both cameras. After optimization, we get good focus on both main and validation cameras. Areas with coated chrome reflect more light resulting in brighter spots on the main camera. The chrome significantly attenuates the forward scattered light, hence they appear as black spots in the validation camera. All images were normalized to the maximum value of the image for visualization purposes, in practice the images with no correction are much darker.

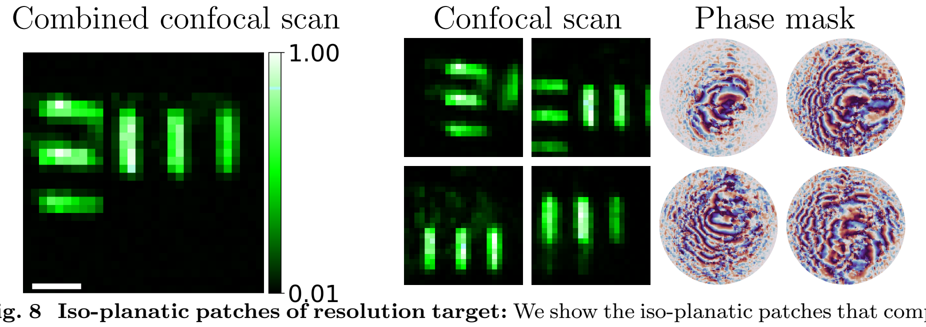

Fig. 8 Iso-planatic patches of resolution target: We show the iso-planatic patches that compose the combined result of the resolution target presented in Fig. 5 in the main paper. The first column shows the result presented in the main paper. The second and third columns show the iso-planatic patches which were scanned by our algorithm. The fourth and fifth columns shows the phase mask used for each iso-planatic patch. The iso-planatic patches were combined using a template matching algorithm.

Onion cells: To better visualize the onion cell target we image in Fig. 7 of the main paper, we present in Fig. 9 a larger view through the validation camera. The final result presented in the main paper in Fig. 7 is too big to be corrected by a single modulation and we have achieved the result by combining multiple isoplanatic patches, stitched to form the large field-of-view image. In Fig. 10, we show the individual isoplanatic patches. The patches partially overlap, and to find the overlap between patches we used template matching. The image in Fig. 7 in the paper was rotated for convenience of view. To understand the typical 3D structure of the onion, in Fig. 11 we show confocal scanning at the front (shallow) parts of the onion where the light is not aberrated.

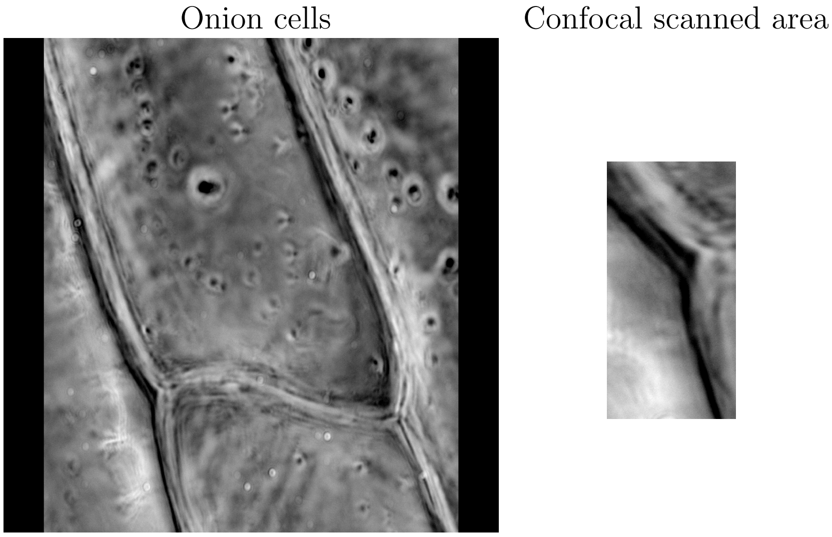

Fig. 9 Onion cells: We show a larger view of the onion cells example from Fig. 7 of the main paper, captured by our validation camera.

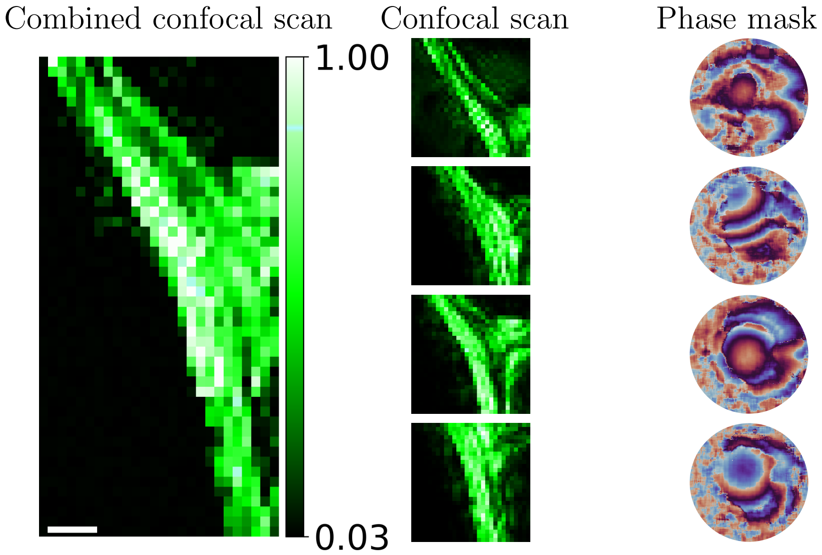

Fig. 10 **Iso-planatic patches in onion result:** We show the iso-planatic patches that compose the combined result of the onion cells boundary presented in Fig. 7 in the main paper. The first column shows the result presented in the main paper. The second column shows the iso-planatic patches which were scanned by our algorithm. The third column shows the phase mask used for each iso-planatic patch. The iso-planatic patches were combined using a template matching algorithm.

In Fig. 12 we show more of the 3D structure results of Fig. 8 of the main paper. For that we include x-y cross sections at multiple depths. We scanned in intervals of $2\mu m$ spanning depth of ± 8 from the focused depth. Layer 1 is at $80\mu m$, layer 2 is at $130\mu m$, and layer 3 is at $190\mu m$.

Comparison with digital correction approaches: In Fig. 13 we further compare our method with the CLASS algorithm [25], a representative digital correction technique [6, 9, 26, 27]. Digital approaches such as CLASS measure the scattered wavefront

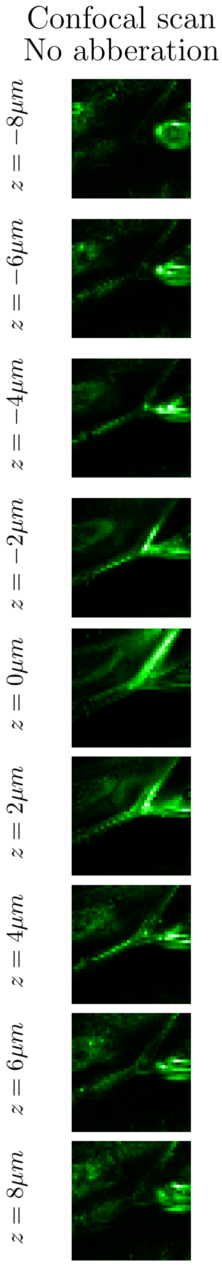

Fig. 11 X-Y cross sections of reference target: To visualize the typical 3D structure of an onion, we show x-y cross sections at multiple depths. This example uses a shallow onion layer without aberration.

and numerically fit it with a parametric model describing the underlying aberration

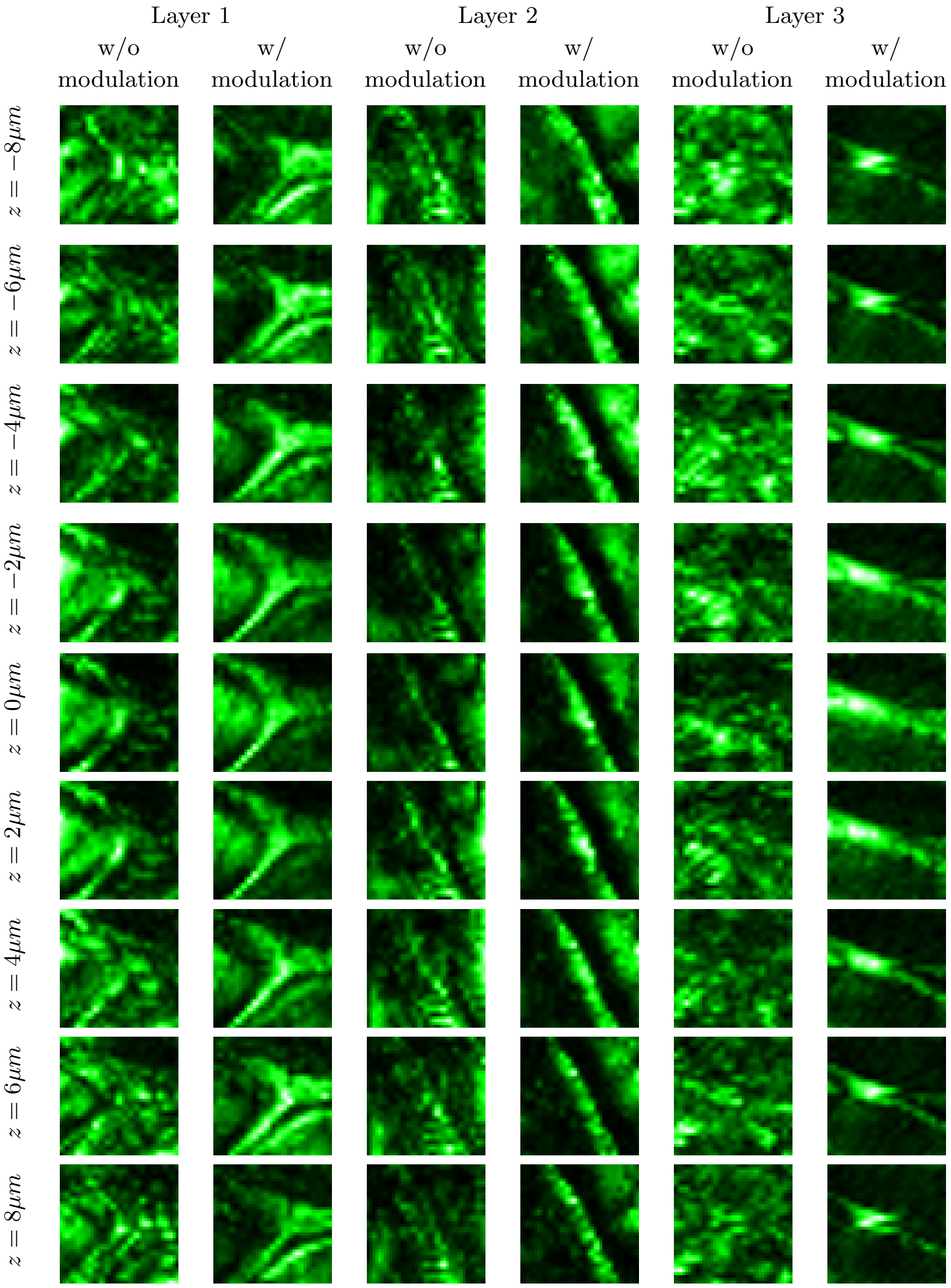

Fig. 12 **X-Y cross sections:** To better visualize the 3D structure, we show additional x-y cross sections of our results of Fig. 8 from the main paper. We scanned in intervals of $2\mu m$ spanning depth of ± 8 from the focused depth. Layer 1 is at $80\mu m$, layer 2 is at $130\mu m$, and layer 3 is at $190\mu m$.

and hidden target. The score function targeted by the CLASS algorithm is similar to the one used in this paper, it attempts to maximize energy along the diagonal of the reflection matrix, which is equivalent to maximizing the confocal energy, as we do in this paper. Because the measured scattered fields are typically very noisy, the reconstruction quality is ultimately limited by the signal-to-noise ratio (SNR) of the acquired data. In contrast, performing the correction *optically*—by applying the estimated modulation directly to the SLM—enhances the effective SNR of subsequent measurements, leading to cleaner reconstructions and improved correction fidelity. In Fig. 13, we visualize CLASS applied to the wavefronts measured at the beginning of our algorithm when the SLM is blank. We then apply CLASS to wavefronts acquired at the fifth and tenth iterations, after placing the correction from the previous iteration on the SLM. With this optical pre-correction, the SNR of the measured data improves substantially, resulting in a markedly better reconstruction.

The CLASS reconstructions shown in Fig. 13 should be regarded as illustrative rather than quantitative benchmarks of CLASS performance, due to two main differences. First, the CLASS system measures the wavefront interferometrically, which can provide more accurate phase recovery than our phase-diversity estimate. Second, the CLASS algorithm typically measures many more columns of the reflection matrix than our system acquires. Reflection-matrix imaging typically scans a large area that covers the speckle support around each corrected point, while our method scans only the corrected region of interest. While this broader sampling can yield more complete information, it also requires scanning a much larger spatial region to cover the speckle support around each corrected point. In contrast, our method scans only the local region of interest, making each acquisition significantly faster, although multiple iterations are then required for convergence.

An interesting future direction would be to combine these two approaches—using the scattered wavefronts measured in the first iteration to fit an aberration model through multiple gradient-descent updates, then placing this model on the SLM and re-measuring data under reduced scattering. Such a hybrid strategy could reduce the number of capture iterations required, although given that our algorithm typically converges within about ten iterations, the expected improvement would likely be limited.

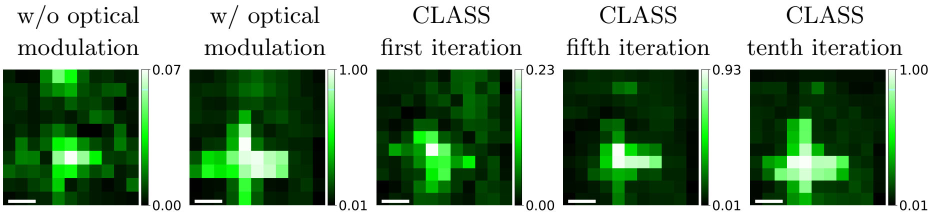

Fig. 13 Comparison to CLASS algorithm: We compare our optical correction approach, with the digital correction CLASS algorithm. We visualize our captured result without applying optical modulation, with applying the optical modulation, and three results of the CLASS algorithm, applied to the wavefronts measured at the beginning of our algorithm when the SLM is blank, and to the wavefronts acquired at the fifth and tenth iterations. Applying optical correction increases the SNR of the measured wavefronts and, as a result, allows the CLASS algorithm to achieve better results.

References

[1] Aizik, D., Levin, A.: Non-invasive and noise-robust light focusing using confocal wavefront shaping. Nature communications **15**(5575) (2024)

[2] Aizik, D., Gkioulekas, I., Levin, A.: Fluorescent wavefront shaping using incoherent iterative phase conjugation. Optica **9**(7), 746–754 (2022)

[3] Mugnier, L.M., Blanc, A., Idier, J.: Phase diversity: A technique for wavefront sensing and for diffraction-limited imaging. Advances in Imaging and Electron Physics, vol. 141, pp. 1–76. Elsevier (2006). https://doi.org/10.1016/S1076-5670(05)41001-0

[4] Najar, U., Barolle, V., Balondrade, P., Fink, M., Boccara, C., Aubry, A.: Harnessing forward multiple scattering for optical imaging deep inside an opaque medium. Nature Communications **15**(1), 7349 (2024) https://doi.org/10.1038/s41467-024-51619-9

[5] Armijo, L.: Minimization of functions having Lipschitz continuous first partial derivatives. Pacific Journal of Mathematics **16**(1), 1–3 (1966)

[6] Balondrade, P., Barolle, V., Guigui, N., Auriant, E., Rougier, N., Boccara, C., Fink, M., Aubry, A.: Multi-spectral reflection matrix for ultrafast 3d label-free microscopy. Nature Photonics **18**(10), 1097–1104 (2024)

[7] Zhang, Y., Dinh, M., Wang, Z., Zhang, T., Chen, T., Hsu, C.W.: Deep imaging inside scattering media through virtual spatiotemporal wavefront shaping. arXiv preprint arXiv:2306.08793 (2024)

[8] Lee, Y., Kim, D., Jo, Y., Kim, M., Choi, W.: Exploiting volumetric wave correlation for enhanced depth imaging in scattering medium. Nature communications **14**(1) (2023)

[9] Kang, S., Kwon, Y., Lee, H., Kim, S., Hong, J., Yoon, S., Choi, W.: Tracing multiple scattering trajectories for deep optical imaging in scattering media. Nature communications **14**(1) (2023)

[10] Katz, O., Small, E., Guan, Y., Silberberg, Y.: Noninvasive nonlinear focusing and imaging through strongly scattering turbid layers. Optica **1**(3), 170–174 (2014)

[11] Osnabrugge, G., Horstmeyer, R., Papadopoulos, I.N., Judkewitz, B., Vellekoop, I.M.: Generalized optical memory effect. Optica **4**(8), 886–892 (2017) https://doi.org/10.1364/optica.4.000886

[12] Alterman, M., Bar, C., Gkioulekas, I., Levin, A.: Imaging with local speckle intensity correlations: theory and practice. ACM TOG (2021)

[13] Dean, B.H., Bowers, C.W.: Diversity selection for phase-diverse phase retrieval.

J. Opt. Soc. Am. A **20**(8), 1490–1504 (2003) https://doi.org/10.1364/JOSAA.20.001490

[14] Gonsalves, R.A.: Phase Retrieval And Diversity In Adaptive Optics. Optical Engineering **21**(5), 215829 (1982) https://doi.org/10.1117/12.7972989

[15] Candes, E.J., Li, X., Soltanolkotabi, M.: Phase retrieval via wirtinger flow: Theory and algorithms. IEEE Transactions on Information Theory **61**(4), 1985–2007 (2015)

[16] Smartt, R.N., Steel, W.H.: Theory and application of point-diffraction interferometers. Japanese Journal of Applied Physics **14**(S1), 351 (1975) https://doi.org/10.7567/JJAPS.14S1.351

[17] Akondi, V., Jewel, A., Vohnsen, B.: Digital phase-shifting point diffraction interferometer. Optics Letters **39**, 1641–4 (2014) https://doi.org/10.1364/OL.39.001641

[18] Song, H.C., Kuperman, W.A., Hodgkiss, W.S., Akal, T., Ferla, C.: Iterative time reversal in the ocean. The Journal of the Acoustical Society of America **105**(6), 3176–3184 (1999) https://doi.org/10.1121/1.424648

[19] Prada, C., Thomas, J.-L., Fink, M.: The iterative time reversal process: Analysis of the convergence. The Journal of the Acoustical Society of America **97**(1), 62–71 (1995) https://doi.org/10.1121/1.412285

[20] Ruan, H., Jang, M., Judkewitz, B., Yang, C.: Iterative time-reversed ultrasonically encoded light focusing in backscattering mode. Scientific reports **4**, 7156 (2014) https://doi.org/10.1038/srep07156

[21] Si, K., Fiolka, R., Cui, M.: Breaking the spatial resolution barrier via iterative sound-light interaction in deep tissue microscopy. Scientific reports **2**, 748 (2012) https://doi.org/10.1038/srep00748

[22] Papadopoulos, I., Jouhanneau, J.-S., Poulet, J., Judkewitz, B.: Scattering compensation by focus scanning holographic aberration probing (f-sharp). Nature Photonics (2016)

[23] Kang, S., Kang, P., Jeong, S., Kwon, Y., Yang, T.D., Hong, J.H., Kim, M., Song, K.-D., Park, J.H., Lee, J.H., Kim, M.J., Kim, K.H., Choi, W.: High-resolution adaptive optical imaging within thick scattering media using closed-loop accumulation of single scattering. Nature Communications **8**(1), 2157 (2017) https://doi.org/10.1038/s41467-017-02117-8

[24] Weise, W., Zinin, P., Wilson, T., Briggs, A., Boseck, S.: Imaging of spheres with the confocal scanning optical microscope. Opt. Lett. **21**(22), 1800–1802 (1996) https://doi.org/10.1364/OL.21.001800

[25] Kang, S., Jeong, S., Choi, W., Ko, H., Yang, T.D., Joo, J.H., Lee, J.-S., Lim, Y.-S., Park, Q.-H., Choi, W.: Imaging deep within a scattering medium using collective accumulation of single-scattered waves. Nature Photonics **9**(4), 253–258 (2015) https://doi.org/10.1038/nphoton.2015.24

[26] Kwon, Y., Hong, J.H., Kang, S., Lee, H., Jo, Y., Kim, K.H., Yoon, S., Choi, W.: Computational conjugate adaptive optics microscopy for longitudinal through-skull imaging of cortical myelin. Nature Communications **14**(1), 105 (2023) https://doi.org/10.1038/s41467-022-35738-9

[27] Haim, O., Boger-Lombard, J., Katz, O.: Image-guided computational holographic wavefront shaping. Nature Photonics (2024)
